# Supplementary material for: Meta-Analysis and Gene Set Enrichment Relative to ER Status Reveal Elevated Activity of MYC and E2F in the “Basal” Breast Cancer Subgroup
Source: PLoS One. 2009 Mar 9;4(3):e4710. doi: 10.1371/journal.pone.0004710 (PMC2650420; doi:10.1371/journal.pone.0004710)
Supplement: Dataset S1 — GSEA results from first screen. The file contains a zipped archive of the HTML results of the second GSEA screen. Reports for enriched or depleted datasets may be accessed from the “index.html” link within the my_analysis.GseaPreranked.1219975950625.rpt.zip ZIP archive. Enrichment plots are available for each of the 50 most enriched and depleted gene sets. (13.32 MB ZIP) [file pone.0004710.s001.zip › my_analysis.GseaPreranked.1219975950625.rpt.zip!//V$E2F1DP1_01.html]

Details for gene set V$E2F1DP1\_01[GSEA]

|  || Dataset | zSco.ERneg.to.ERpos\_collapsed |
| Phenotype | NoPhenotypeAvailable |
| Upregulated in class | na\_pos |
| GeneSet | V$E2F1DP1\_01 |
| Enrichment Score (ES) | 0.48708504 |
| Normalized Enrichment Score (NES) | 2.142943 |
| Nominal p-value | 0.0 |
| FDR q-value | 7.565308E-5 |
| FWER p-Value | 0.0030 |
Table: GSEA Results Summary

  

Fig 1: Enrichment plot: V$E2F1DP1\_01      
 Profile of the Running ES Score & Positions of GeneSet Members on the Rank Ordered List

  

| PROBE | GENE SYMBOL | GENE\_TITLE | RANK IN GENE LIST | RANK METRIC SCORE | RUNNING ES | CORE ENRICHMENT || 1 | E2F3 |  |  | 6 | 7.369 | 0.0201 | Yes |
| 2 | NASP |  |  | 59 | 6.201 | 0.0334 | Yes |
| 3 | PIM1 |  |  | 78 | 6.025 | 0.0488 | Yes |
| 4 | AK2 |  |  | 85 | 5.971 | 0.0650 | Yes |
| 5 | GPRC5B |  |  | 103 | 5.845 | 0.0800 | Yes |
| 6 | MCM4 |  |  | 180 | 5.436 | 0.0894 | Yes |
| 7 | MCM6 |  |  | 198 | 5.317 | 0.1029 | Yes |
| 8 | GMNN |  |  | 206 | 5.302 | 0.1171 | Yes |
| 9 | PAX6 |  |  | 224 | 5.208 | 0.1304 | Yes |
| 10 | HMGA1 |  |  | 244 | 5.125 | 0.1432 | Yes |
| 11 | CDC25A |  |  | 281 | 4.993 | 0.1544 | Yes |
| 12 | MCM7 |  |  | 289 | 4.970 | 0.1677 | Yes |
| 13 | STMN1 |  |  | 310 | 4.907 | 0.1798 | Yes |
| 14 | EZH2 |  |  | 312 | 4.888 | 0.1934 | Yes |
| 15 | ARHGAP11A |  |  | 361 | 4.763 | 0.2030 | Yes |
| 16 | RANBP1 |  |  | 380 | 4.733 | 0.2148 | Yes |
| 17 | SYNCRIP |  |  | 402 | 4.679 | 0.2263 | Yes |
| 18 | SNRPD1 |  |  | 440 | 4.552 | 0.2361 | Yes |
| 19 | DMD |  |  | 481 | 4.406 | 0.2454 | Yes |
| 20 | SUV39H1 |  |  | 495 | 4.361 | 0.2565 | Yes |
| 21 | TYRO3 |  |  | 516 | 4.316 | 0.2671 | Yes |
| 22 | RPS20 |  |  | 615 | 4.101 | 0.2710 | Yes |
| 23 | MCM2 |  |  | 630 | 4.080 | 0.2813 | Yes |
| 24 | MCM3 |  |  | 656 | 4.019 | 0.2906 | Yes |
| 25 | NCL |  |  | 678 | 3.977 | 0.3001 | Yes |
| 26 | TMPO |  |  | 792 | 3.788 | 0.3021 | Yes |
| 27 | ILF3 |  |  | 801 | 3.774 | 0.3120 | Yes |
| 28 | MSH2 |  |  | 819 | 3.748 | 0.3212 | Yes |
| 29 | MYC |  |  | 1025 | 3.447 | 0.3152 | Yes |
| 30 | UNG |  |  | 1070 | 3.390 | 0.3213 | Yes |
| 31 | FANCC |  |  | 1175 | 3.264 | 0.3225 | Yes |
| 32 | NOLC1 |  |  | 1222 | 3.217 | 0.3280 | Yes |
| 33 | PRKDC |  |  | 1234 | 3.204 | 0.3360 | Yes |
| 34 | DNAJC9 |  |  | 1243 | 3.198 | 0.3443 | Yes |
| 35 | CDC2 |  |  | 1259 | 3.182 | 0.3521 | Yes |
| 36 | POLD1 |  |  | 1260 | 3.182 | 0.3609 | Yes |
| 37 | CDC5L |  |  | 1278 | 3.162 | 0.3685 | Yes |
| 38 | POU4F1 |  |  | 1296 | 3.142 | 0.3759 | Yes |
| 39 | FBXO5 |  |  | 1304 | 3.137 | 0.3841 | Yes |
| 40 | SFRS10 |  |  | 1320 | 3.118 | 0.3917 | Yes |
| 41 | PCNA |  |  | 1346 | 3.087 | 0.3984 | Yes |
| 42 | ING3 |  |  | 1398 | 3.042 | 0.4030 | Yes |
| 43 | EFNA5 |  |  | 1488 | 2.953 | 0.4045 | Yes |
| 44 | ATAD2 |  |  | 1532 | 2.917 | 0.4093 | Yes |
| 45 | PRPS2 |  |  | 1592 | 2.859 | 0.4128 | Yes |
| 46 | EPHB1 |  |  | 1675 | 2.780 | 0.4143 | Yes |
| 47 | NUP62 |  |  | 1684 | 2.770 | 0.4214 | Yes |
| 48 | E2F1 |  |  | 1695 | 2.764 | 0.4284 | Yes |
| 49 | HIRA |  |  | 1696 | 2.763 | 0.4361 | Yes |
| 50 | PKMYT1 |  |  | 1715 | 2.753 | 0.4424 | Yes |
| 51 | FKBP5 |  |  | 1729 | 2.743 | 0.4490 | Yes |
| 52 | SUMO1 |  |  | 1736 | 2.736 | 0.4562 | Yes |
| 53 | PLAGL1 |  |  | 1762 | 2.718 | 0.4619 | Yes |
| 54 | UGCGL1 |  |  | 1779 | 2.709 | 0.4682 | Yes |
| 55 | CASP8AP2 |  |  | 1930 | 2.587 | 0.4640 | Yes |
| 56 | RRM2 |  |  | 2047 | 2.485 | 0.4621 | Yes |
| 57 | H2AFZ |  |  | 2105 | 2.447 | 0.4646 | Yes |
| 58 | MAP3K7 |  |  | 2143 | 2.421 | 0.4685 | Yes |
| 59 | SFRS7 |  |  | 2226 | 2.370 | 0.4689 | Yes |
| 60 | POLR2A |  |  | 2274 | 2.334 | 0.4719 | Yes |
| 61 | FANCG |  |  | 2282 | 2.332 | 0.4778 | Yes |
| 62 | GLRA3 |  |  | 2348 | 2.287 | 0.4793 | Yes |
| 63 | SOAT1 |  |  | 2362 | 2.281 | 0.4846 | Yes |
| 64 | IPO7 |  |  | 2413 | 2.247 | 0.4871 | Yes |
| 65 | HNRPD |  |  | 2585 | 2.156 | 0.4801 | No |
| 66 | PTMA |  |  | 2647 | 2.119 | 0.4814 | No |
| 67 | DNMT1 |  |  | 2844 | 2.017 | 0.4721 | No |
| 68 | ATF5 |  |  | 2941 | 1.962 | 0.4703 | No |
| 69 | NR6A1 |  |  | 3000 | 1.931 | 0.4713 | No |
| 70 | RASAL2 |  |  | 3067 | 1.901 | 0.4715 | No |
| 71 | CDC6 |  |  | 3185 | 1.847 | 0.4678 | No |
| 72 | BRPF1 |  |  | 3197 | 1.841 | 0.4721 | No |
| 73 | C10ORF119 |  |  | 3202 | 1.838 | 0.4769 | No |
| 74 | MAPK6 |  |  | 3396 | 1.751 | 0.4671 | No |
| 75 | PAQR4 |  |  | 3434 | 1.736 | 0.4692 | No |
| 76 | PRPF4B |  |  | 3435 | 1.736 | 0.4740 | No |
| 77 | SMAD6 |  |  | 3436 | 1.736 | 0.4788 | No |
| 78 | TOPBP1 |  |  | 3475 | 1.722 | 0.4807 | No |
| 79 | PPIG |  |  | 3502 | 1.707 | 0.4835 | No |
| 80 | SLCO3A1 |  |  | 3563 | 1.679 | 0.4836 | No |
| 81 | MSH5 |  |  | 3656 | 1.639 | 0.4812 | No |
| 82 | SLC9A7 |  |  | 3790 | 1.589 | 0.4755 | No |
| 83 | PRPS1 |  |  | 3889 | 1.556 | 0.4724 | No |
| 84 | POLE2 |  |  | 3975 | 1.520 | 0.4702 | No |
| 85 | RQCD1 |  |  | 4253 | 1.412 | 0.4531 | No |
| 86 | HNRPUL1 |  |  | 4310 | 1.387 | 0.4527 | No |
| 87 | GRIA4 |  |  | 4341 | 1.377 | 0.4543 | No |
| 88 | CLSPN |  |  | 4483 | 1.324 | 0.4472 | No |
| 89 | GABRB3 |  |  | 4549 | 1.303 | 0.4459 | No |
| 90 | KIAA0101 |  |  | 4770 | 1.230 | 0.4327 | No |
| 91 | STAG1 |  |  | 4794 | 1.225 | 0.4343 | No |
| 92 | CORT |  |  | 4877 | 1.196 | 0.4314 | No |
| 93 | HNRPR |  |  | 4913 | 1.185 | 0.4321 | No |
| 94 | HTF9C |  |  | 4986 | 1.161 | 0.4298 | No |
| 95 | ARHGAP6 |  |  | 4991 | 1.159 | 0.4328 | No |
| 96 | MYH10 |  |  | 5076 | 1.124 | 0.4295 | No |
| 97 | EIF4A1 |  |  | 5195 | 1.083 | 0.4236 | No |
| 98 | HIST1H4A |  |  | 5241 | 1.067 | 0.4231 | No |
| 99 | EED |  |  | 5267 | 1.060 | 0.4242 | No |
| 100 | CCNT1 |  |  | 5647 | 0.931 | 0.3980 | No |
| 101 | ACO2 |  |  | 5713 | 0.907 | 0.3956 | No |
| 102 | SEMA6A |  |  | 5935 | 0.836 | 0.3811 | No |
| 103 | PB1 |  |  | 5977 | 0.822 | 0.3803 | No |
| 104 | ADAMTS2 |  |  | 5988 | 0.817 | 0.3818 | No |
| 105 | YBX2 |  |  | 6299 | 0.716 | 0.3602 | No |
| 106 | ALDH6A1 |  |  | 6317 | 0.711 | 0.3609 | No |
| 107 | C20ORF67 |  |  | 6482 | 0.657 | 0.3503 | No |
| 108 | H2AFV |  |  | 6569 | 0.627 | 0.3455 | No |
| 109 | SPTB |  |  | 6641 | 0.609 | 0.3418 | No |
| 110 | POLD3 |  |  | 6667 | 0.601 | 0.3416 | No |
| 111 | TFAP4 |  |  | 6777 | 0.561 | 0.3349 | No |
| 112 | ZIM2 |  |  | 7151 | 0.436 | 0.3078 | No |
| 113 | AP1S1 |  |  | 7316 | 0.377 | 0.2964 | No |
| 114 | GATA1 |  |  | 7431 | 0.334 | 0.2886 | No |
| 115 | MGC13053 |  |  | 7620 | 0.261 | 0.2751 | No |
| 116 | TBX6 |  |  | 7723 | 0.223 | 0.2679 | No |
| 117 | SP3 |  |  | 7914 | 0.141 | 0.2539 | No |
| 118 | PEG3 |  |  | 8018 | 0.099 | 0.2464 | No |
| 119 | SLC9A5 |  |  | 8303 | 0.000 | 0.2248 | No |
| 120 | MXD3 |  |  | 8312 | -0.002 | 0.2242 | No |
| 121 | HIST1H2BK |  |  | 8356 | -0.020 | 0.2210 | No |
| 122 | HMG2L1 |  |  | 8412 | -0.042 | 0.2169 | No |
| 123 | SYNGR4 |  |  | 8638 | -0.147 | 0.2002 | No |
| 124 | FMO4 |  |  | 8775 | -0.199 | 0.1904 | No |
| 125 | SFRS1 |  |  | 8891 | -0.255 | 0.1824 | No |
| 126 | DCK |  |  | 8935 | -0.280 | 0.1799 | No |
| 127 | ID3 |  |  | 8993 | -0.308 | 0.1765 | No |
| 128 | PPM1D |  |  | 9078 | -0.350 | 0.1711 | No |
| 129 | FLJ25476 |  |  | 9347 | -0.481 | 0.1520 | No |
| 130 | PCSK1 |  |  | 9351 | -0.483 | 0.1532 | No |
| 131 | MAZ |  |  | 9415 | -0.521 | 0.1498 | No |
| 132 | HIST1H3A |  |  | 9503 | -0.563 | 0.1448 | No |
| 133 | ZCCHC8 |  |  | 9511 | -0.566 | 0.1458 | No |
| 134 | HNRPA2B1 |  |  | 9531 | -0.578 | 0.1460 | No |
| 135 | FLJ10081 |  |  | 9711 | -0.675 | 0.1343 | No |
| 136 | ASXL2 |  |  | 9736 | -0.689 | 0.1344 | No |
| 137 | ZCWPW1 |  |  | 9773 | -0.709 | 0.1336 | No |
| 138 | GSPT1 |  |  | 9841 | -0.751 | 0.1306 | No |
| 139 | MRPL40 |  |  | 10273 | -1.004 | 0.1007 | No |
| 140 | XTP3TPA |  |  | 10592 | -1.236 | 0.0800 | No |
| 141 | C11ORF30 |  |  | 10656 | -1.284 | 0.0788 | No |
| 142 | DLG3 |  |  | 10923 | -1.502 | 0.0627 | No |
| 143 | C14ORF130 |  |  | 10945 | -1.523 | 0.0654 | No |
| 144 | AP4M1 |  |  | 11123 | -1.683 | 0.0566 | No |
| 145 | CSPG2 |  |  | 11207 | -1.748 | 0.0552 | No |
| 146 | PHF15 |  |  | 11215 | -1.755 | 0.0595 | No |
| 147 | HOXC10 |  |  | 11345 | -1.878 | 0.0550 | No |
| 148 | FHOD1 |  |  | 11408 | -1.941 | 0.0557 | No |
| 149 | USP52 |  |  | 11931 | -2.478 | 0.0229 | No |
| 150 | CXORF12 |  |  | 12144 | -2.762 | 0.0145 | No |
| 151 | RPS6KA5 |  |  | 12285 | -2.949 | 0.0121 | No |
| 152 | NELL2 |  |  | 12593 | -3.420 | -0.0017 | No |
| 153 | MAPT |  |  | 12626 | -3.480 | 0.0056 | No |
| 154 | RET |  |  | 12638 | -3.506 | 0.0145 | No |
| 155 | ARID4A |  |  | 12806 | -3.906 | 0.0127 | No |
| 156 | APPL |  |  | 13059 | -4.678 | 0.0066 | No |
| 157 | TLE3 |  |  | 13070 | -4.720 | 0.0190 | No |
Table: GSEA details [plain text format]

  

Fig 2: V$E2F1DP1\_01: Random ES distribution      
 Gene set null distribution of ES for **V$E2F1DP1\_01**
